# Nutritional intake and anthropometric characteristics are associated with endurance performance and markers of low energy availability in young female cross-country skiers

**DOI:** 10.1080/15502783.2023.2226639

**Published:** 2023-06-21

**Authors:** Oona Kettunen, Ritva Mikkonen, Vesa Linnamo, Jaakko Mursu, Heikki Kyröläinen, Johanna K. Ihalainen

**Affiliations:** Faculty of Sport and Health Sciences, University of Jyväskylä, Vuokatti, Finland

**Keywords:** Carbohydrate, fat, macronutrient, protein, sports nutrition, VO_2max_

## Abstract

**Background:**

Low energy availability (LEA) can have negative performance consequences, but the relationships between LEA and performance are poorly understood especially in field conditions. In addition, little is known about the contribution of macronutrients to long-term performance. Therefore, the aim of this study was to evaluate if energy availability (EA) and macronutrient intake in a field-based situation were associated with laboratory-measured performance, anthropometric characteristics, blood markers, training volume, and/or questionnaire-assessed risk of LEA in young female cross-country (XC) skiers. In addition, the study aimed to clarify which factors explained performance.

**Methods:**

During a one-year observational study, 23 highly trained female XC skiers and biathletes (age 17.1 ± 1.0 years) completed 3-day food and training logs on four occasions (September–October, February–March, April–May, July–August). Mean (±SD) EA and macronutrient intake from these 12 days were calculated to describe yearly overall practices. Laboratory measurements (body composition with bioimpedance, blood hormone concentrations, maximal oxygen uptake (VO_2max_), oxygen uptake (VO_2_) at 4 mmol·L^−1^ lactate threshold (OBLA), double poling (DP) performance (time to exhaustion), counter movement jump (height) and the Low Energy Availability in Females Questionnaire (LEAF-Q)) were completed at the beginning (August 2020, M_1_) and end of the study (August 2021, M_2_). Annual training volume between measurements was recorded using an online training diary.

**Results:**

The 12-day mean EA (37.4 ± 9.1 kcal·kg FFM^−1^·d^−1^) and carbohydrate (CHO) intake (4.8 ± 0.8 g·kg^−1^·d^−1^) were suboptimal while intake of protein (1.8 ± 0.3 g·kg^−1^·d^−1^) and fat (31 ± 4 E%) were within recommended ranges. Lower EA and CHO intake were associated with a higher LEAF-Q score (*r*  = 0.44, *p*  = 0.042; *r*  = 0.47, *p*  = 0.026). Higher CHO and protein intake were associated with higher VO_2max_ (*r*  = 0.61, *p*  = 0.005; *r*  = 0.54, *p*  = 0.014), VO_2_ at OBLA (*r*  = 0.63, *p*  = 0.003; *r*  = 0.62, *p*  = 0.003), and DP performance at M_2_ (*r*  = 0.42, *p*  = 0.051; *r*  = 0.44, *p*  = 0.039). Body fat percentage (F%) was negatively associated with CHO and protein intake (*r* = -0.50, *p*  = 0.017; *r* = -0.66, *p*  = 0.001). Better DP performance at M_2_ was explained by higher training volume (R^2^  = 0.24, *p*  = 0.033) and higher relative VO_2max_ and VO_2_ at OBLA at M_2_ by lower F% (R^2^  = 0.44, *p*  = 0.004; R^2^  = 0.47, *p*  = 0.003). Increase from M_1_ to M_2_ in DP performance was explained by a decrease in F% (R^2^  = 0.25, *p*  = 0.029).

**Conclusions:**

F%, and training volume were the most important factors explaining performance in young female XC skiers. Notably, lower F% was associated with higher macronutrient intake, suggesting that restricting nutritional intake may not be a good strategy to modify body composition in young female athletes. In addition, lower overall CHO intake and EA increased risk of LEA determined by LEAF-Q. These findings highlight the importance of adequate nutritional intake to support performance and overall health.

## 1. Introduction

The relationship between energy availability (EA) and performance is not yet fully understood, although the negative consequences of low energy availability (LEA) on health are quite well documented, especially with females [1]. Most importantly, there is a significant gap in knowledge between short-term laboratory studies and long-term field-based studies examining LEA [2]. Although actual cutoff values are individual, LEA is often defined as < 30 kcal·kg FFM^−1^·d^−1^ based on laboratory studies in sedentary women [3]. LEA may suppress endocrine function, impair bone health, and increase the risk of illness while leaving inadequate amounts of energy for recovery and training adaptations, which all have negative consequences for performance development [1,2,4]. Changes in hormone levels, menstrual disturbances, and increased incidence of bone stress injuries are typical symptoms and signs of LEA [1,2,4]. Regrettably, relatively little is known about EA in cross country (XC) skiers who are prone to several LEA risk factors such as high exercise energy expenditure (EEE) [5] and the performance benefits of lean body composition (i.e. more fat free mass (FFM) and less fat mass (FM)) [6].

In addition to EA, the macronutrient composition of a diet can also have a significant impact on performance and health. Carbohydrates (CHO), in particular, are known to be a critical macronutrient for performance in endurance sports such as XC skiing [5,7]. Indeed, XC ski races and key training sessions are conducted at intensities that mostly depend on CHO based fuels [5,8]. To maintain capacity for high working intensities between hard training sessions and competitions, consumption of high amounts of CHO is necessary [9]. Unfortunately, previous studies have shown that both elite [10] and young female skiers [11] struggle to meet the current CHO recommendations for endurance athletes. In contrast, the recommendations for protein and fat intake are met by most XC skiers [10,11], which supports recovery, training adaptations, and normal body functions [12].

Females, and especially females under 20 years of age, who compete in endurance and/or lean sports (e.g. running, XC skiing), are especially prone to LEA associated health and performance problems [1,13]. Regrettably, most studies that have focused on the macronutrient requirements in athletes have been conducted with male participants resulting in a significant gap in knowledge about macronutrient needs in female athletes [14]. Therefore, the aim of this study was to investigate whether EA and macronutrient intake in field-based conditions are associated with the changes in performance, anthropometric characteristics, blood markers, training volume, and/or questionnaire-determined risk for LEA. In addition, the study aimed to clarify which of the above-mentioned variables explained maximal oxygen uptake (VO_2max_), lactate threshold, upper body endurance performance, and lower limb explosive performance, i.e. the factors that contribute to performance in XC skiing [15].

## 2. Materials and methods

### 2.1. Participants

A total of 27 female XC skiers and biathletes (age 17.1 ± 1.0 years, VO_2max_ 54.1 ± 3.8 ml·kg^−1^·min^−1^) participated in the study. All participants provided informed consent after receiving comprehensive oral and written details of the protocol. The participants were members of the sport academy of a local high school. Four participants dropped out due to personal reasons and thus the final number of the participants was 23. Altogether 64% of the participants belonged to Youth National Teams. The Ethical Board of the University of Jyväskylä approved the study procedures (No. 380/13.00.04.00/2020), and the study was conducted in accordance with the Declaration of Helsinki.

### 2.2. Design

The one-year follow-up study was performed from August 2020 (M_1_) to August 2021 (M_2_) to examine whether 12-day mean EA and macronutrient intake assessed from four 3-day food and training logs in different phases of the training year were associated with changes in performance, anthropometric characteristics, and serum hormone concentrations in young female XC skiers. The Low Energy Availability in Females Questionnaire (LEAF-Q) [16] and laboratory measurements including performance tests, anthropometric measurements, and blood samples were completed at the beginning (M_1_) and at the end of the study (M_2_). In addition, anthropometric measurements and aerobic performance tests were performed twice during the follow-up (November and the following April) to refine EA assessment (see calculations). Annual training volume was recorded using an online training diary (eLogger, eSportwise Oy, Finland).

### 2.3. Anthropometric measurements

Anthropometric measurements were completed in the morning following an overnight fast. Height of the participants was measured with a wall-mounted stadiometer. Body mass (BM), FFM, FM, and fat percentage (F%) were measured using bioimpedance (Inbody 720, Biospace Co., Seoul, Korea).

### 2.4. Blood samples and analysis

Fasting blood samples were obtained from the antecubital vein for analysis of serum hormone concentrations. M_1_ and M_2_ samples from each participant were collected at the same time of day between 7 a.m. and 9 a.m. Blood was drawn into Vacuette gel serum tubes (Greiner-Bio-One GmbH, Kremsmünster, Austria). The tubes were centrifuged at 3600 rpm for 10 min to collect serum, which was frozen at -20°C. Concentrations of insulin, cortisol, insulin-like growth factor 1 (IGF-1), free triiodothyronine (T3), free thyroxine (T4), thyroid-stimulating hormone (TSH), leptin, and testosterone were analyzed by an immunometric chemiluminescence method (Immulite 2000 ×Pi, Siemens Healthcare, United Kingdom). The assay sensitivities were 2.0 U·L^−1^ for insulin, 5.5 nmol·L^−1^ for cortisol, 2.6 nmol·L^−1^ for IGF-1, 1.5 pmol·L^−1^ for free T3, 1.4 for free T4, 0.004 mU·L^−1^ for TSH, 0.2 ug·L^−1^ for leptin, and 0.5 nmol·L^−1^ for testosterone. Reliabilities expressed as a coefficient of variation were 8.8% for insulin, 7.7% for cortisol, 4.4% for IGF-1, 10.3% for free T3, 6.6% for free T4, 9.2% for TSH, 4.9% for leptin, and 10.9% for testosterone.

### 2.5. Jump performance

The counter movement jump (CMJ) test [17] was performed on a force plate (HUR FP8, Kokkola, Finland) after a self-selected warm-up. Participants were instructed to stand with feet shoulder-width apart and hands on hips while flexing the knees and trying to jump as high as possible. The best jump height of three attempts was calculated from impulse [18].

### 2.6. Aerobic capacity and lactate threshold

The incremental aerobic capacity test, which was familiar to the participants, was performed by walking or running with poles on a treadmill (Telineyhtymä, Kotka, Finland) ∼15 min after CMJ. The test started at 3.5° inclination with a speed of 5.0 km·h^−1^. The inclination and/or the speed of the treadmill was increased every third minute so that predicted oxygen uptake (VO_2_) calculated according to the equation by Balke & Ware [19] increased by 6 ml·kg^−1^·min^−1^ in every stage.

Respiratory variables were measured continuously using a mixing chamber system (Medikro 919 Ergospirometer, Medikro Oy, Kuopio, Finland). Volume and gas calibration of the ergospirometer was done prior to every measurement. VO_2_ and respiratory exchange ratio (RER) from the last 60 s of each stage, and the highest 60 s average (VO_2max_) were recorded. Heart rate was monitored continuously throughout the tests using a heart rate belt (Polar H10, Polar Electro Oy, Kempele, Finland), and the average heart rate from the last 60 s of each stage was recorded. Blood lactate samples at the end of each stage were obtained from a fingertip and collected into capillary tubes (20 μL), which were placed in a 1-mL hemolyzing solution and analyzed using Biosen C-line analyzer (EKF diagnostics, Barleben, Germany). In addition to VO_2max_, the VO_2_ at 4 mmol·L^−1^ lactate (Onset of Blood Lactate Accumulation, OBLA) was recorded.

### 2.7. Double poling performance

A maximal double poling test was performed by roller skiing on a treadmill (Rodby Innovation AB, Vänge, Sweden). All skiers used the same pair of Marwe 800 ×C roller skis (Marwe Oy, Hyvinkää, Finland) equipped with prolink bindings (Salomon Group, Annecy, France) and standard 6C6 wheels (Marwe Oy, Hyvinkää, Finland). Poles (Swix Triac 3.0, BRAV, Lillehammer, Norway) were selected based on the length of the participants´ classic poles and were equipped with a tip customized for treadmill roller skiing. After a self-selected warm-up outside the laboratory, participants performed a 10 min warm-up at the same workload as the first stage of the subsequent submaximal test including two ∼15 s sprints at the workload equal to the fourth to sixth stage of the test. Following the warm-up, athletes performed an incremental treadmill test double poling at an incline of 2°, starting at 10 km·h^−1^ and followed by an increase of 1 km·h^−1^ every minute until volitional exhaustion. Time to exhaustion was also recorded.

### 2.8. Food and training logs

Food and training logs were collected at four time points (September–October, February–March, April–May, July–August) for a 3-day period. Athletes selected three subsequent days for each log from a 4-week period, and the mean values from 12 days were calculated to describe yearly overall practices. Participants were asked to select days that described their typical daily dietary and training routines as well as possible. Timing, amount, and type of food as well as fluid consumed were recorded in the food logs while calibrated kitchen scales were used to weigh servings. If the scales were not available (i.e. in a restaurant), participants took photos of their portions. They were also asked to take at least two photos of the weighted portions to validate what was recorded. Timing, type, and average heart rate of exercises performed were recorded in training logs. Written and verbal instructions were given to ensure more accurate record keeping. Food logs were analyzed for energy intake (EI) and macronutrient intake using Aivodiet-software (version 2.0.2.3, Mashie, Malmö, Sweden). All dietary records were analyzed by the same researcher. Despite limitations in validity of food logs, they are currently the best available tool to assess dietary intake of the athletes in the field conditions [20].

### 2.9. Calculations

The EEE assessments were based on the individual relationship of energy expenditure (EE), oxygen uptake, and heart rate during the aerobic capacity test. EE during the first five stages of the test was calculated as follows:

EE = VO_2_ * (1.1 * RER + 3.9) [21]

Calculated EE and heart rate from the five first stage of the test were used to form an individual regression line for each subject as described by Tomten & Hostmark [22]. EEE of each exercise was calculated from the mean heart rate and duration of the training session using this regression line. As the relationship between EE and heart rate may change within a year, the aerobic capacity test was performed four times during the study (August, November, April, August) to update the regression line. The Cunningham equation [23], utilizing FFM from the bioimpedance measurement, was used to calculate resting EE, which was subtracted from EEE to follow the latest definition of EA [24]. Laboratory-based measurements, where heart rate is plotted against indirect calorimetry are among the most valid methods to assess EEE in field conditions [2].

Daily EA was calculated as:

EA = (EI – EEE) / FFM [3],

where FFM is obtained from bioimpedance measurement performed within ∼a month of the logs.

To enable a comprehensive understanding of the nutritional practices during the whole training year, the 12-day mean EI, EEE, EA, and macronutrient intake was used in the analysis.

### 2.10. Questionnaires

Participants completed the LEAF-Q [16] at the beginning and at the end of the study, which was used to assess the risk of LEA (≥8 points [16]) and the prevalence of self-reported amenorrhea (absence of menstrual cycles for more than 90 d [25]). The LEAF-Q consist of questions regarding physiological symptoms linked to energy deficiency, such as injuries, gastrointestinal symptoms, and menstrual dysfunction [16].

### 2.11. Statistical analysis

IBM SPSS Statistics version 26.0 (IBM Corp., Armonk, NY) was used for statistical analysis. A Shapiro–Wilk test was performed to check the normality of the data. Nonparametric tests were used to analyze non-normally distributed data (FFM at M_1_, BM at M_2_, FM at M_2_). The significance of the changes from M_1_ to M_2_ were analyzed either with Student´s paired t-test (normally distributed data) or Wilcoxon signed rank test (non-normally distributed data). Results are reported as means ± SD. The effect size of differences was expressed as Cohen’s *d* [26]. Correlations for normally distributed data were analyzed by using Pearson’s correlation coefficient (*r*) while nonparametric data was analyzed using Kendall’s tau b (τ_b_). Stepwise linear regression analysis was used to determine which of the nutritional, anthropometric, and training volume variables presented in Table 3 explained performance variables in the end of the follow-up period (M_2_). BM and FM were excluded from the regression analyses because the normality of the variables was violated. The method selected the best variables to explain the dependent variable. In the analysis of year-to-year changes in the performance variables, the changes in body composition, instead of absolute values, were used as independent variables. Homoscedasticity was tested by estimating Pearson and Spearman’s correlations between the standardized predicted values and the absolute standardized residuals and was not violated in any of the analyses. The relative importance of contributing variables was calculated based on R Square (R^2^). The statistical significance was defined as *p* < .05.

**Table 1.** The 12-day means for energy intake, exercise energy expenditure, energy availability, and macronutrient intake in young female cross-country skiers.

*n* = 23	Mean ± SD	Range	Recommendation [12]
Energy intake (kcal·kg FFM^−1^·d^−1^)	50.3 ± 7.7	32.2–63.0	NA
Exercise energy expenditure (kcal·kg FFM^−1^·d^−1^)	12.8 ± 4.5	7.5–20.9	NA
Energy availability (kcal·kg FFM^−1^·d^−1^)	37.4 ± 9.1	12.4–49.0	≥45
Carbohydrate intake (g·kg^−1^·d^−1^)	4.8 ± 0.8	2.8–6.4	6–10
Protein intake (g·kg^−1^·d^−1^)	1.8 ± 0.3	1.2–2.4	1.2–2.0
Fat intake (E%)	31 ± 4	24–37	20–35 E%

**Table 2.** Anthropometric characteristics, performance variables, hormone concentrations, blood lipids, and questionnaire scores in young female cross-country skiers in two measurement point (M_1_, M_2_) with one year interval (Mean ± SD). Significance of the differences between M_1_ and M_2_ is presented in *p* values and effect sizes (Cohen’s d.).

	n	M_1_	M_2_	*p*	*d*
**Anthropometrics**					
Body mass (kg)	22	61.8 ± 7.1	64.5 ± 7.7	<.001	0.90
Height	22	168.4 ± 5.1	169.1 ± 5.1	.024	0.52
BMI (kg·m^−2^)	22	21.8 ± 2.2	22.4 ± 2.3	.001	0.78
Fat free mass (kg)	22	51.2 ± 5.2	52.6 ± 4.9	<.001	0.75
Fat mass (kg)	22	10.6 ± 3.4	11.9 ± 4.3	.038	0.44
Fat percentage (%)	22	17.0 ± 4.3	18.1 ± 4.9	.10	0.37
**Performance**					
VO_2max_ (L·min^−1^)	20	3.3 ± 0.3	3.3 ± 0.3	.41	0.18
VO_2max_ (ml·kg^−1^·min^−1^)	20	54.1 ± 3.8	52.1 ± 4.3	.006	0.70
VO_2_ at OBLA (L·min^−1^)	20	2.9 ± 0.3	2.9 ± 0.3	.45	0.17
VO_2_ at OBLA (ml·kg^−1^·min^−1^)	20	47.1 ± 3.4	46.4 ± 4.2	.26	0.24
DP (s)	22	492 ± 68	494 ± 70	.91	0.03
CMJ (cm)	21	30.9 ± 3.8	30.7 ± 4.1	.69	0.09
**Hormone concentrations**					
Insulin (mU·L^−1^)	23	8.9 ± 6.6	7.9 ± 4.3	.44	0.17
Cortisol (nmol·L^−1^)	23	554 ± 150	591 ± 161	.21	0.27
IGF-1 (nmol·L^−1^)	23	34.6 ± 10.0	36.0 ± 12.1	.57	0.12
Free T3 (pmol·L^−1^)	23	4.7 ± 1.1	4.6 ± 1.0	.50	0.14
Free T4 (pmol·L^−1^)	23	13.5 ± 2.4	13.2 ± 1.8	.66	0.09
TSH (mU·L^−1^)	23	2.5 ± 1.4	2.4 ± 0.9	.73	0.07
Leptin (ng·L^−1^)	23	24.3 ± 13.8	30.1 ± 15.9	.21	0.26
Testosterone (nmol·L^−1^)	23	0.8 ± 0.4	0.7 ± 0.3	.47	0.15
**Questionnaires**					
LEAF-Q	22	5.5 ± 3.6	7.3 ± 4.5	.08	0.40

BMI, body mass index; VO_2max_, maximal oxygen uptake; VO_2_ at OBLA, oxygen uptake in 4.0 mmol·l^−1^ lactate level; DP time from double poling test; CMJ, counter movement jump on the force plate; IGF-1, insulin-like growth factor 1; T3, triiodothyronine; T4, thyroxine; TSH, thyroid-stimulating hormone; LEAF-Q, The Low Energy Availability in Females Questionnaire. Statistically significant *p* values are bolded.

**Table 3.** Correlation coefficients (r or τ_b_) between EA, macronutrient intake, and performance variables at the end of the follow-up period (M_2._).

	VO_2max_ (L·min^−1^)	VO_2max_ (ml·kg^−1^·min^−1^)	VO_2_ at OBLA (L·min^−1^)	VO_2_ at OBLA (ml·kg^−1^·min^−1^)	DP (s)	CMJ (cm)
EA (kcal·kg FFM^−1^·d^−1^) (*r*)	-0.36	0.31	-0.27	0.32	.27	0.22
CHO (g·kg^−1^·d^−1^) (*r*)	-0.21	0.61**	-0.10	0.63**	.42	0.39
Protein (g·kg^−1^·d^−1^) (*r*)	0.02	0.54*	0.15	0.62**	.44*	0.29
Fat (g·kg^−1^·d^−1^) (*r*)	-0.24	0.42	-0.07	0.53*	.26	0.23
Body mass (kg) (τ_b_)	0.41*	-0.46**	0.42**	-0.41*	-.09	-0.28
Body mass index (kg·m^−2^) (*r*)	0.74***	-0.40	0.64**	-0.40	.13	-0.50*
Fat free body mass (kg) (*r*)	0.76***	-0.37	0.72***	-0.31	.09	-0.27
Body fat mass (kg) (τ_b_)	0.15	-0.52**	0.20	-0.45**	-.23	-0.27
Fat percentage (%) (*r*)	0.24	-0.67**	0.21	-0.61**	-.38	-0.37
Training volume (h·y^−1^) (*r*)	0.57*	0.43	0.39	0.22	.41*	0.04

EA, energy availability; CHO, carbohydrate intake; VO2max, maximal oxygen uptake; VO2 at OBLA, oxygen uptake in 4.0 mmol·l-^1^ lactate level; DP, time from double poling test; CMJ, counter movement jump on the force plate; r, variable analyzed using Pearson´s r; τb, a nonparametric variable that is analyzed by using Kendall’s tau-b; * significant correlation p < .05; ** p < .01. Statistically significant correlations are bolded.

## 3. Results

Table 1 presents the mean EI, EEE, EA, and macronutrient intake from the food and training logs (the mean of 12 d). The mean EA (37.4 ± 9.1 kcal·kg FFM^−1^·d^−1^) and CHO (4.8 ± 0.8 g·kg^−1^·d^−1^) intake were below the recommendation while protein (1.8 ± 0.3 g·kg^−1^·d^−1^) and fat intake (1.4 ± 0.4 g·kg^−1^·d^−1^) were at recommended range.

BM, height, body mass index (BMI), FFM, FM, and F% increased significantly during the follow-up year (Table 2), while VO_2max_ in relation to BM decreased (Table 2). The results of other performance variables and blood hormone concentrations remained similar between M_1_ and M_2_. Nine athletes had LEAF-Q score ≥ 8 at both measurement points. Five of the athletes had score of ≥ 8 at both M_1_ and M_2_, four athletes only in M_1_, and four athletes only in M_2_. One subject had self-reported amenorrhea at both M_1_ and M_2_. This participant had LEA (26.4 kcal·kg FFM^−1^·d^−1^), a high LEAF-Q score (12 at M_1_ and 15 at M_2_), and her concentrations of several metabolic hormones were below the lower limit of 95% confidence interval (CI) for the group mean. Namely, concentrations of insulin at M_1_ (4.0, CI: 5.8–11.5) free T3 at M_1_ (3.7 pmol·l^−1^, CI: 4.2–5.2 pmol·l^−1^) and at M_2_ (3.0 pmol·l^−1^, CI: 4.2–5.1 pmol·l^−1^), free T4 at M_1_ (10.0 pmol·l^−1^, CI: 12.5–14.5 pmol·l^−1^) and at M_2_ (8.2 pmol·l^−1^, CI: 12.5–14.0 pmol·l^−1^), TSH at M_1_ (0.9 mU·l^−1^, CI: 1.9–3.1) and at M_2_ (1.1 mU·l^−1^, CI: 2.0–2.8 mU·l^−1^), and leptin at M_2_ (14.2 ng·L^−1^, CI: 23.2–37.0 ng·L^−1^) were below 95% CI.

On average, participants trained 601 ± 101 (454–811) hours during the follow-up year. Training volume was not associated with anthropometric or nutritional variables. Participants who had higher training volume generally performed better in the DP test at M_2_ (*r* = 0.49, *p* = 0.033) and had higher absolute VO_2max_ (L·min^−1^) at M_2_ (*r* = 0.57, *p* = 0.18). In addition, training volume showed a statistical trend toward higher relative VO_2max_ (ml·kg^−1^·min^−1^) at M_2_ (*r* = 0.42, *p* = 0.092), and toward increase in DP test time (*r* = 0.43, *p* = 0.069) and relative VO_2max_ (*r* = 0.40, *p* = 0.11) from M_1_ to M_2_.

**Figure 1.**
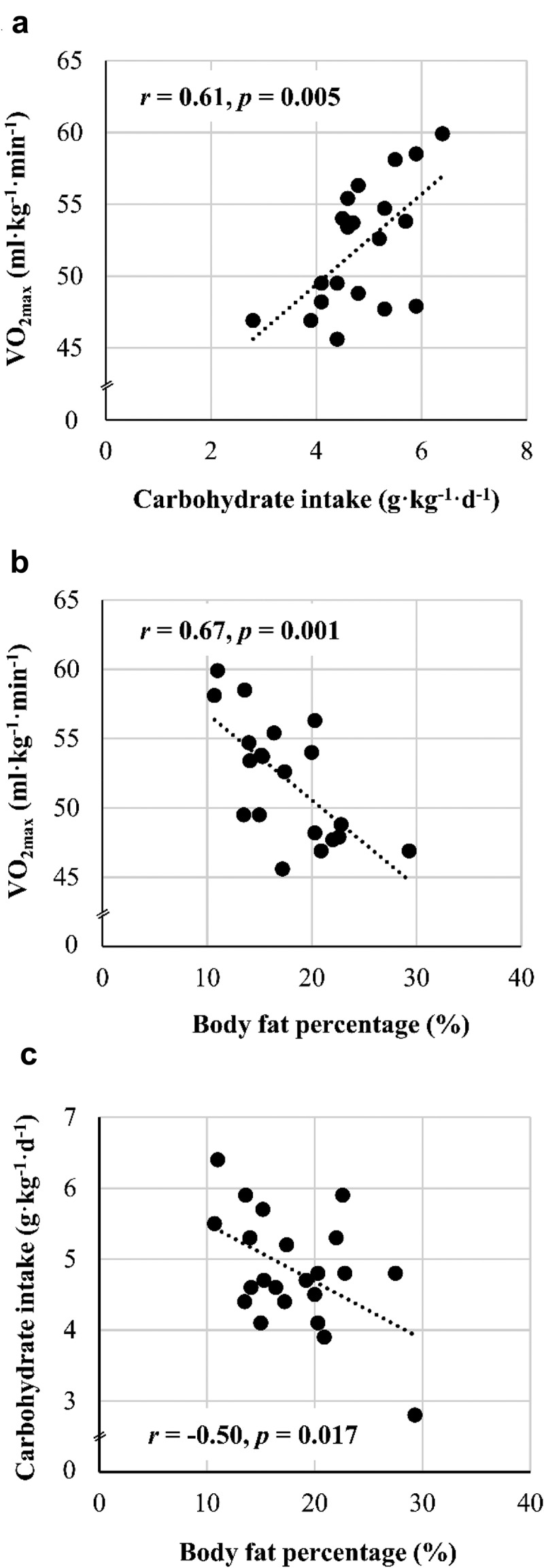
Associations between carbohydrate intake and maximal oxygen uptake (VO_2max_) (A), body fat percentage and VO_2max_ (B), and body fat percentage and carbohydrate intake.

Table 3 shows the correlations between performance, nutrition, body composition, and training volume. Athletes with higher relative VO_2max_ and VO_2_ at OBLA at M_2_ tended to consume more macronutrients, particularly CHO (Figure 1a) and protein, in relation to their BM. In addition, CHO and protein intake were positively associated with the DP test time, but in the case of CHO, this association did not reach statistical significance (*p* = 0.051). The only positive association between performance changes and nutrition was the positive association between protein intake and the change from M_1_ to M_2_ in relative VO_2_ at OBLA (*r* = 0.55, *p* = 0.013). As shown in Table 3, absolute VO_2max_ and VO_2_ at OBLA were positively associated with BM, BMI, and FFM while relative VO_2max_ and VO_2_ at OBLA were negatively associated with BM, FM, and F% (Figure 1b). DP test time did not correlate with anthropometric variables. CMJ was negatively associated with BMI. Training volume was positively associated with absolute VO_2max_ and DP test time (Table 3).

BM at M_2_ was negatively associated with CHO intake (τ_b_ = 0.33, *p* = 0.036), FM at M_2_ with protein intake (τ_b_ = 0.43, *p* = 0.006) and F% at M_2_ with both CHO (Figure 1c) and protein intake (*r* = -0.50, *p* = 0.017; *r* = -0.66, *p* = 0.001, respectively). There were no associations between nutritional intake and changes in anthropometric characteristics or between nutritional intake and hormone concentrations. In contrast, EA and CHO intake were negatively associated with the LEAF-Q score at M_2_ (*r* = 0.44, *p* = 0.042; *r* = 0.47, *p* = 0.026, respectively).

**Figure 2.**
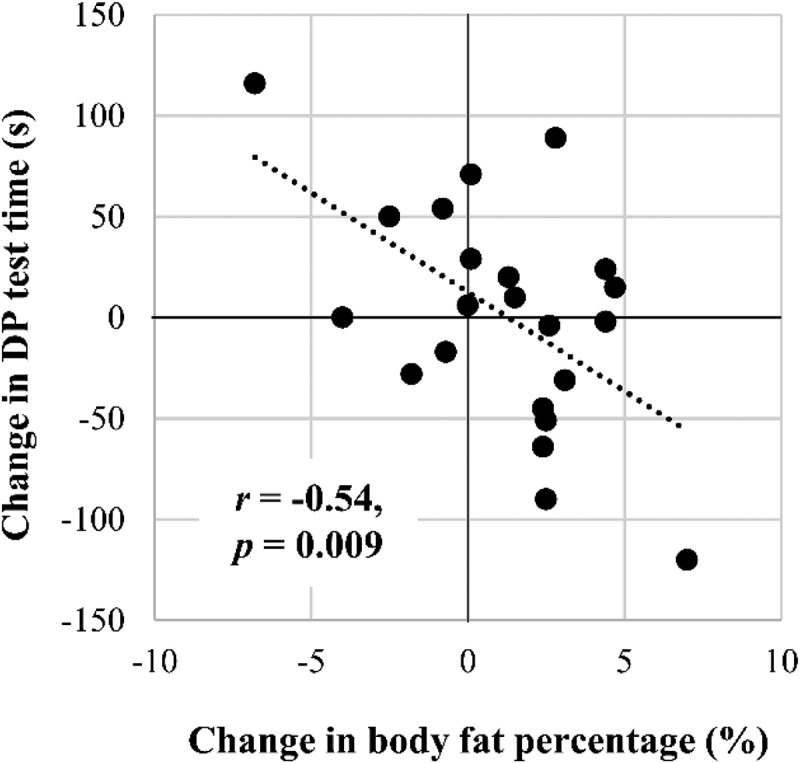
Association between changes in body fat percentage and double poling (DP) test time.

In the stepwise linear regression analysis, DP test time at M_2_ was explained by training volume (R^2^ = 0.24, *p* = 0.033), absolute VO_2max_ by BMI and training volume (R^2^ = 0.71, *p* < .001), relative VO_2max_ (ml·kg^−1^·min^−1^) by F% (R^2^ = 0.44, *p* = 0.004), absolute VO_2_ at OBLA by FFM (R^2^ = 0.64, *p* < .001), and relative VO_2_ at OBLA by F% (R^2^ = 0.47, *p* = 0.003). Changes from M_1_ to M_2_ in the DP test time were explained by changes in F% (R^2^ = 0.25, *p* = 0.029, Figure 2). Changes in VO_2max_, VO_2_ at OBLA, or CMJ were not explained by training volume, nutrition, or anthropometric characteristics.

## 4. Discussion

The present study aimed to clarify how 12-day mean EA and macronutrient intake in field-based situations are associated with performance, anthropometric characteristics, hormonal concentrations, LEAF-Q points, and/or training volume. The results indicated that young female XC skiers with higher CHO and protein intake had higher VO_2max_ and VO_2_ at OBLA in relation to their BM as well as better DP performance. Nevertheless, anthropometric characteristics and higher training volume seemed to be the most important factors explaining endurance performance. The results also indicated that lower EA and CHO intake were associated with higher LEA risk (high LEAF-Q score).

The 12-day mean EA (37.4 ± 9.1 kcal·kg FFM^−1^·d^−1^) was at a suboptimal level in light of the EA recommendation for female athletes [3]. In addition, four (17 %) of the participants had LEA. These values are comparable to those previously reported from a shorter follow-up period [11]. On average, the young female XC skiers appear to have EA that is above the “historical” LEA threshold of 30 kcal·kg FFM^−1^·d^−1^ [3]. Notably, this threshold is based on the short-term laboratory interventions, that showed impaired endocrine [27] and bone turnover markers [28]. Unfortunately, the minimum EA level that can be maintained over longer periods (weeks, months, or years) without negative effects on health and performance is currently unknown and likely individual [2]. Therefore, we do not know with certainty whether the mean EA observed in the present study ultimately affects long-term health and/or performance.

EA assessment in field conditions have several methodological challenges that can reduce the validity of the observations [2,29]. Consequently, researchers have recommended the use of surrogate markers of LEA (i.e. blood parameters and/or questionnaires) for screening purposes [2,29]. As such, the assessment of LEAF-Q scores and blood hormone analyses were included in the study protocol. As expected, lower EA was associated with a higher risk of LEA determined by LEAF-Q score, and indeed, all of the athletes with LEA, as determined by food diaries, had a LEAF-Q score ≥ 8 at the end of the follow-up period. These findings suggest that food and training logs may provide important information regarding the risk of LEA if the recording period is long enough.

Blood hormone concentrations remained similar from M_1_ to M_2_ and were not associated with other variables. This could be due to the fact that hormonal disturbances are typically reported as a result of LEA [27,30], which was avoided by most participants in the present study. Indeed, the only amenorrheic athlete had insulin, free T3, free T4, TSH, and leptin concentrations that were among the lowest of the entire group (below the lower limit of 95% CI). Previous studies have shown decreased insulin, free T3, and leptin are associated with LEA and amenorrhea, while the results for free T4 and TSH are variable [30]. In addition to decreased levels of metabolic hormones, the amenorrheic participant was identified as having LEA determined by food and training logs and was classified as high risk for LEA based on LEAF-Q. This case suggests that food and training logs, LEAF-Q, and measurement of specific hormones could all be successfully used for screening purposes in athletes with long-term LEA. Nevertheless, this conclusion is limited because we did not perform a clinical diagnosis to exclude other possible causes of amenorrhea. In addition, CVs of the hormone concentrations were relatively high and thus the interpretations should be made with caution.

As in previous studies in female XC skiers [10,11], CHO intake (4.8 ± 0.8 g·kg^−1^·d^−1^) was lower than recommended for endurance athletes training 1–3 h per day (6–10 g·kg^−1^·d^−1^ [12]), while protein (1.8 ± 0.3 g·kg^−1^·d^−1^), and fat intake (31 ± 4 of total EI) were within the recommended range (protein 1.2–2.0 g·kg^−1^·d^−1^; fat 20–35% of total EI [12]). Interestingly, athletes with higher CHO and protein intake tended to have better relative VO_2max_, VO_2_ at OBLA, and DP test time in the end of the follow-up period, which suggest that eating practices that include higher amounts of these macronutrients might be beneficial for long-term performance adaptations. This finding is not surprising as the intensities used in XC skiing competitions and key training sessions are reliant on CHO based fuels [5,8], and relatively high CHO intake is needed to replenish muscle glycogen stores between training sessions [9]. In addition, adequate CHO intake is important to minimize the risk of illness [31] and overtraining [32]. Protein, in turn, plays an essential role as a substrate for recovery and trigger for adaptation after exercise [33], thus adequate amount of protein is needed for recovery and training adaptation. Given that high load endurance training [34], whole body training [35], adolescence [36], energy deficiency [37] and low CHO availability [38] may increase protein requirements, a protein intake in the upper range or slightly above current recommendations [12] may have been beneficial for the participants in the present study.

Despite the associations between nutrition and performance, the strongest factors explaining better performance were related to leaner body composition and higher training volume. Training volume during the one-year follow-up explained 24% of the variation in the DP test time at the end of the study, while 25% of the annual changes of the DP test time were explained by changes in F%. This is consistent with the findings of Jones et al. (2021), who reported that changes toward leaner body composition favor the development in XC ski performance [6]. In particular, increased upper-body strength and lean mass can lead to improved DP performance [6,39]. In addition, it is known that a higher training volume is an important factor that differentiates elite XC skiers from national level athletes [40], which highlights the importance of high training volume for performance development.

Lower F% was beneficial for relative VO_2max_ and explained 44% of the variation, while F% alone explained 47% of the variation in relative VO_2_ at OBLA. Although mean relative VO_2max_ and VO_2_ at OBLA decreased and BM, FM, and F% increased during the follow-up, changes in VO_2max_ and VO_2_ at OBLA could not be explained by changes in anthropometric characteristics. Interestingly, lower BM, FM, and F% were associated with higher EA and macronutrient intake but there were no associations between nutrition and changes in anthropometric characteristics. Together these findings suggest that associations between nutrition, anthropometric characteristics, and maximal and submaximal aerobic performance have likely developed over a longer period of time than what was investigated. Nonetheless, the better performing athletes tended to have higher macronutrient intakes, which may have promoted their development over the years. Notably, the results of this study suggest that restricting EA or macronutrient intake is not an effective way to modify BM and/or body composition in young female endurance athletes. In addition, the results highlight that the adequacy of an athletes´ dietary intake cannot be determined based on BM or body composition [1,41,42].

Although problems with the validity and reliability of assessing dietary intake and EA in field conditions [2,29] can be considered as the main limitation of the present study, the methods used to minimize methodological errors were the strength of the study. As real-world EA assessment has been criticized as a short recording period only gives a snapshot of an athlete’s dietary and training practices [2,19], the present study aimed to provide an overall picture of nutritional and training practices by analyzing a total of 12 days of nutrition and training data from different parts of the training year. Although misreporting may limit the validity of the food logs, comprehensive written and verbal instructions, weighted food logs and highly motivated participants increased the validity of the present data. To increase the validity of EEE assessment, laboratory-based associations of EE, VO_2_, and heart rate were utilized [2]. Unfortunately, for practical reasons, we could not measure body composition using the gold standard method DXA and the use of the less precise bioimpedance method is a minor limitation of the study. To minimize potential confounding factors, the measurement was always performed after an overnight fast and after the participants had visited the toilet. In addition, we were not able to standardize the phase of the menstrual cycle, which may have influenced on the bioimpedance analysis as well as on the other variables investigated. Additionally, while this study lacks assessment of competition performance, all performance parameters included are considered important for assessing XC skier development [15]. Furthermore, a recent study by Talsnes et al. (2021) showed that laboratory-based VO_2max_ incremental running test and roller ski performance tests have a strong positive correlation with competition performance [43].

## 5. Conclusions

Anthropometric characteristics, especially F%, and training volume appeared to be the most important factors explaining different indicators of a young female XC skier’s performance. In addition, lower CHO and protein intake were associated with higher BM, FM, and F% as well as with lower VO_2max_ and DP performance. Taken together, these findings suggest that although lean body composition may be beneficial for endurance performance, it should not be sought by restricting dietary intake. Indeed, athletes with lower CHO intake and EA not only had poorer performance, but also an increased risk of self-reported physiological symptoms of LEA. These findings highlight the importance of adequate EA and macronutrient intake to support performance and overall health.

## Data analysis

Conceptualization and methodology, OK, VL, JKI; investigation, OK; statistical analysis, OK, JM; original draft preparation, OK; Writing – review and editing: OK, RM, VL, JM, HK, JKI; supervision RM, VL, JKI. All authors have read and agreed to the published version of the manuscript.

## References

[1] Mountjoy, M, Sundgot-Borgen, JK, Burke, LM, et al. IOC consensus statement on relative energy deficiency in sport (RED-S): 2018 update. Br J Sports Med. 2018;52(11):687–519. doi: 10.1136/bjsports-2018-099193 29773536

[2] Heikura, IA, Stellingwerff, T, Areta, JL. Investigating the effect of bouncing type on the physiological demands of trampolining. Eur J Sport Sci. 2021;21(1):1–6. doi: 10.1080/17461391.2020.1721564 31973686

[3] Loucks, AB, Kiens, B, Wright, HH. Energy availability in athletes. J Sports Sci. 2011;29(sup1):S7–S15. doi: 10.1080/02640414.2011.588958 21793767

[4] Logue, DM, Madigan, SM, Melin, A, et al. Low energy availability in athletes 2020: an updated narrative review of prevalence, risk, within-day energy balance, knowledge, and impact on sports performance. Nutr. 2020;12(3):1–19. doi: 10.3390/nu12030835 PMC7146210 32245088

[5] Heikura, IA, Kettunen, O, Garthe, I, et al. Energetic demands and nutritional strategies of elite cross-country skiers during tour de Ski: a narrative review. J Sci Sport Exerc. 2021;3(3):224–237. doi: 10.1007/s42978-020-00105-x

[6] Jones, TW, Lindblom, HP, Karlsson, Ø, et al. Anthropometric, physiological, and performance developments in cross-country skiers. Med Sci Sports Exercise. 2021;53(12):2553–2564. doi: 10.1249/MSS.0000000000002739 34649265

[7] Burke, LM, Hawley, JA, Wong, SHS, et al. Carbohydrates for training and competition carbohydrates for training and competition. J Sports Sci. 2011;29(sup1):S17–S27. doi: 10.1080/02640414.2011.585473 21660838

[8] Hawley, JA, Leckey, JJ. Carbohydrate dependence during prolonged, intense endurance exercise. Sports Med. 2015;45(S1):5–12. doi: 10.1007/s40279-015-0400-1 PMC4672006 26553495

[9] Burke, LM, Van Loon, LJC, Hawley, JA. Postexercise muscle glycogen resynthesis in humans. J Appl Physiol. 2017;122(5):1055–1067. doi: 10.1152/japplphysiol.00860.2016 27789774

[10] Carr, A, Mcgawley, K, Govus, A, et al. Nutritional intake in elite cross-country skiers during two days of training and competition. Int J Sport Nutr Exerc Metab. 2019;29(3):273–281. doi: 10.1123/ijsnem.2017-0411 29989466

[11] Kettunen, O, Heikkilä, M, Linnamo, V, et al. Nutrition knowledge is associated with energy availability and carbohydrate intake in young female cross-country skiers. Nutr. 2021;13(6):1769. doi: 10.3390/nu13061769 PMC8224650 34067303

[12] Thomas, DT, Erdman, KA, Burke, LM. American college of sports medicine joint position statement. Nutrition and athletic performance. Med Sci Sports Exercise. 2016;48(3):543–568. doi: 10.1249/MSS.0000000000000852 26891166

[13] Nose-Ogura, S, Yoshino, O, Dohi, M, et al. Risk factors of stress fractures due to the female athlete triad: differences in teens and twenties. Scand J Med Sci Sports. 2019;29(10):1501–1510. doi: 10.1111/sms.13464 31100189

[14] Moore, DR, Sygo, J, Morton, JP. Fuelling the female athlete: carbohydrate and protein recommendations. Eur J Sport Sci. 2021;22(5):684–696. doi: 10.1080/17461391.2021.1922508 34015236

[15] Sandbakk, Ø, Holmberg, HC. Physiological capacity and training routines of elite cross-country skiers: approaching the upper limits of human endurance. Int J Sports Physiol Perform. 2017;12(8):1003–1011. doi: 10.1123/ijspp.2016-0749 28095083

[16] Melin, A, Tornberg, ÅB, Skouby, S, et al. The LEAF questionnaire: a screening tool for the identification of female athletes at risk for the female athlete triad. Br J Sports Med. 2014;48(7):540–545. doi: 10.1136/bjsports-2013-093240 24563388

[17] Bosco, C, Mognoni, P, Luhtanen, P. Relationship between isokinetic performance and ballistic movement. Eur J Appl Physiol. 1983;51(3):357–364. doi: 10.1007/BF00429072 6685034

[18] Linthorne, NP. Analysis of standing vertical jumps using a force platform. Am J Phys. 2001;69(11):1198–1204. doi: 10.1119/1.1397460

[19] Balke, B, Ware, R. An experimental study of physical fitness of air force personnel. U S Armed Forces Med J. 1959;10:675–688. 13659732

[20] Capling, L, Beck, KL, Gifford, JA, et al. Validity of dietary assessment in athletes: a systematic review. Nutr. 2017;9(12):1313. doi: 10.3390/nu9121313 PMC5748763 29207495

[21] Weir, JB. New methods for calculating metabolic rate with special reference to protein metabolism. J Physiol. 1949;109(1–2):1–9. doi: 10.1113/jphysiol.1949.sp004363 15394301 PMC1392602

[22] Tomten, SE, Høstmark, AT. Energy balance in weight stable athletes with and without menstrual disorders. Scand J Med Sci Sports. 2006;16(2):127–133. doi: 10.1111/j.1600-0838.2005.00451.x 16533351

[23] Cunningham, JJ. Body composition as a determinant of energy expenditure: a synthetic review and a proposed general prediction equation. Am J Clinic Nutr. 1991;54(6):963–969. doi: 10.1093/ajcn/54.6.963 1957828

[24] Areta, JL, Taylor, HL, Koehler, K. Low energy availability: history, definition and evidence of its endocrine, metabolic and physiological effects in prospective studies in females and males. Eur J Appl Physiol. 2021;121(1):1–21. doi: 10.1007/s00421-020-04516-0 33095376 PMC7815551

[25] Nattiv, A, Loucks, AB, Manore, MM, et al. The female athlete triad. Med Sci Sports Exercise. 2007;39:1867–1882. 10.1249/mss.0b013e318149f111 17909417

[26] Cohen, J. Statistical power analysis for the behavioral sciences. 2nd ed. Hilsdale, NJ: Lawrence Earlbaum Associates; 1988.

[27] Loucks, AB, Thuma, JR. Luteinizing hormone pulsatility is disrupted at a threshold of energy availability in regularly menstruating women. J Clin Endocrinol Metab. 2003;88(1):297–311. doi: 10.1210/jc.2002-020369 12519869

[28] Ihle, R, Loucks, AB. Dose-response relationships between energy availability and bone turnover in young exercising women. J Bone Miner Res. 2004;19(8):1231–1240. doi: 10.1359/JBMR.040410 15231009

[29] Burke, LM, Lundy, B, Fahrenholtz, IL, et al. Pitfalls of conducting and interpreting estimates of energy availability in free-living athletes. Int J Sport Nutr Exerc Metab. 2018;28(4):350–363. doi: 10.1123/ijsnem.2018-0142 30029584

[30] Elliott-Sale, KJ, Tenforde, AS, Parziale, AL, et al. Endocrine effects of relative energy deficiency in sport. Int J Sport Nutr Exerc Metab. 2018;28(4):335–349. doi: 10.1123/ijsnem.2018-0127 30008240

[31] Nieman, DC, Mitmesser, SH. Potential impact of nutrition on immune system recovery from heavy exertion: a metabolomics perspective. Nutr. 2017;9(5):513–523. doi: 10.3390/nu9050513 PMC5452243 28524103

[32] Meeusen, R, Duclos, M, Foster, C, et al. Prevention, diagnosis, and treatment of the overtraining syndrome: joint consensus statement of the European college of sport science and the American college of sports medicine. Med Sci Sports Exercise. 2013;45:186–205. 10.1249/MSS.0b013e318279a10a 23247672

[33] Phillips, SM, van Loon, LJC. Dietary protein for athletes: from requirements to optimum adaptation. J Sports Sci. 2011;29(sup1):S29–S38. doi: 10.1080/02640414.2011.619204 22150425

[34] Churchward-Venne, TA, Pinckaers, PJM, Smeets, JSJ, et al. Dose-response effects of dietary protein on muscle protein synthesis during recovery from endurance exercise in young men: a double-blind randomized trial. Am J Clin Nutr. 2020:1–15. 10.1093/ajcn/nqaa073 PMC7398777 32359142

[35] Macnaughton, LS, Wardle, SL, Witard, OC, et al. The response of muscle protein synthesis following whole-body resistance exercise is greater following 40 g than 20 g of ingested whey protein. Physiol Rep. 2016;4(15):1–13. doi: 10.14814/phy2.12893 PMC4985555 27511985

[36] Desbrow, B, Burd, NA, Tarnopolsky, M, et al. Nutrition for special populations: young, female, and masters athletes. Int J Sport Nutr Exerc Metab. 2019;29(2):220–227. doi: 10.1123/ijsnem.2018-0269 30632423

[37] Areta, JL, Burke, LM, Camera, DM, et al. Reduced resting skeletal muscle protein synthesis is rescued by resistance exercise and protein ingestion following short-term energy deficit. Am J Physiol Endocrinol Metab. 2014;306(8):E989–E997. doi: 10.1152/ajpendo.00590.2013 24595305

[38] Gillen, JB, West, DWD, Williamson, EP, et al. Low-carbohydrate training increases protein requirements of endurance athletes. Med Sci Sports Exercise. 2019;51(11):2294–2301. doi: 10.1249/MSS.0000000000002036 31083047

[39] Hegge, AM, Bucher, E, Ettema, G, et al. Gender differences in power production, energetic capacity and efficiency of elite cross-country skiers during whole-body, upper-body, and arm poling. Eur J Appl Physiol. 2016;116(2):291–300. doi: 10.1007/s00421-015-3281-y 26476546

[40] Sandbakk, Ø, Hegge, AM, Losnegard, T, et al. The physiological capacity of the world’s highest ranked female cross-country skiers. Med Sci Sports Exercise. 2016;48(6):1091–1100. doi: 10.1249/MSS.0000000000000862 PMC5642331 26741124

[41] Vanheest, JL, Rodgers, CD, Mahoney, CE, et al. Ovarian suppression impairs sport performance in junior elite female swimmers. Med Sci Sports Exercise. 2014;46(1):156–166. doi: 10.1249/MSS.0b013e3182a32b72 23846160

[42] Heikura, IA, Stellingwerff, T, Bergland, D, et al. Low energy availability is difficult to assess but outcomes have large impact on bone injury rates in elite distance athletes. Int J Sport Nutr Exerc Metab. 2018;28(4):403–411. doi: 10.1123/ijsnem.2017-0313 29252050

[43] Talsnes, RK, Solli, GS, Kocbach, J, et al. Laboratory- and field-based performance-predictions in cross-country skiing and roller-skiing. Plos One. 2021;16(8):1–17. doi: 10.1371/journal.pone.0256662 PMC8384222 34428258

